# Single ring isolation of pulmonary veins combined with electrical isolation of the superior vena cava in patients with paroxysmal atrial fibrillation

**DOI:** 10.3389/fcvm.2022.1028053

**Published:** 2023-01-09

**Authors:** Xi Li, Yanhong Chen, Guanghui Chen, Chengang Deng, Chen Tang, Jinlin Zhang

**Affiliations:** Department of Cardiology, Wuhan Asian Heart Hospital, Wuhan, Hubei, China

**Keywords:** ablation, catheter, pulmonary vein isolation, paroxysmal atrial fibrillation, superior vena cava isolation, common ostium of pulmonary veins, single ring isolation, left atrial posterior wall isolation

## Abstract

**Background:**

Single-ring isolation (SRI) of the pulmonary veins and the left atrial post wall (LAPW) is an accepted strategy in atrial fibrillation ablation. Whether SRI combined with superior vena cava isolation (SVCI) could further increase the success rate of paroxysmal atrial fibrillation (PAF) has not been reported.

**Objective:**

This study aimed to investigate whether SRI combined with SVCI was feasible and whether it could improve the success rate of PAF ablation.

**Methods and results:**

In our study, sixty patients with PAF from May 2019 to March 2021 were included. SRI plus SVCI was completed with ablation index (AI)-guided high-power ablation. The success rates of SRI and SVCI were 100% and 97%, respectively. One-pass SRI was achieved in 41 out of 60 patients, with 19 out of 60 patients requiring additional ablation to complete the SRI. SVC was not isolated in 2 out of the 60 cases due to concerns about the phrenic nerve (PN) injury. Among the enrolled patients, 2 patients had anomalous pulmonary veins (PVs) (common ostium of inferior PVs). SRI was applied to achieve the PV and PW isolation. After ablation, one patient had an ischemic stroke but recovered without severe morbidity. The average follow-up period was (20 ± 7) months, and single-procedure freedom from atrial arrhythmia was 91.7%. AT/AF recurred in five patients, and 2 out of 5 patients underwent redo ablation. The multi-procedure freedom from atrial arrhythmia was 95%.

**Conclusion:**

Our novel ablation strategy, SRI combined with SVCI, in patients with PAF was feasible and safe, with a relatively high success rate.

## Introduction

The muscle sleeves around the pulmonary veins (PVs) are an established source of ectopic beats, initiating frequent paroxysms of atrial fibrillation (AF) (1). Pulmonary vein isolation (PVI) is the cornerstone of catheter ablation of AF (2). Commonly, two separate rings around the ipsilateral PVs are used (3, 4). However, the non-pulmonary vein (PV) ectopic beats initiating PAF should also be taken into account, especially the left atrial posterior wall (LAPW) (5). Previous studies reported that single-ring isolation (SRI) for paroxysmal and persistent AF and long-term freedom from arrhythmia were comparable to other AF ablation techniques (6–8). In addition, the superior vena cava (SVC) has been shown as one of the most important sites of non-PV AF triggers, and SVC isolation (SVCI), in addition to PV isolation, reduces the recurrence of AF (9–13). SRI plus SVCI in PAF has not been reported, and whether such an ablation strategy could improve the success rate of catheter ablation of PAF is unknown.

The aim of this study was to investigate the safety and feasibility of this ablation strategy for PAF and its success rate.

## Methods

###  Patient selection

From May 2019 to March 2021, 60 patients with PAF were included in our study. PAF was defined as a sustained episode lasting≦7 days. The exclusion criteria included valvular AF, history of cardiac surgery, dialysis or heart failure (LVEF < 30% and New York Heart Association classification III or IV), left atrial thrombus, and age over 80 years. Written informed consent was obtained from all patients. All data were analyzed retrospectively and anonymously. Our study protocol has been approved by the Wuhan Asian Heart Hospital Ethical Committee.

### General principles of ablation

**Figure 1:**
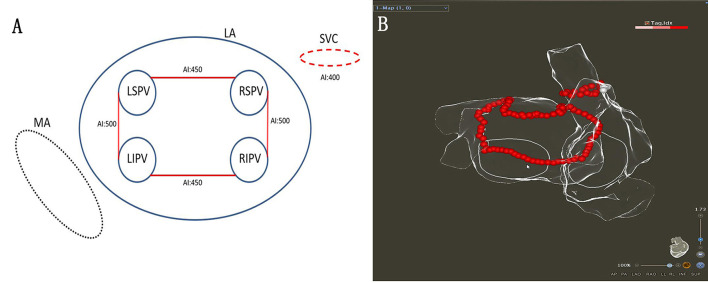
**(A,B)** Schematic figure of ablation strategy used in this study.

The procedure was performed under general anesthesia using the CARTO 3 System (Biosense Webster, Diamond Bar, CA). A decapolar coronary sinus (CS) catheter was introduced *via* the left subclavian vein. When two atrial septal punctures were completed, the following two catheters were introduced *via* the femoral vein: (1) an irrigated-tip radiofrequency (RF) ablation catheter (ThermoCool SmartTouch STSF; Biosense Webster) and (2) a multipolar mapping catheter (Pentaray; Biosense Webster). The target activated clotting time was 300–350 s. A left atrial 3D electroanatomical map using the CARTO 3 System was obtained with the Pentaray^®^ catheter. The linear ablation was completed under AI-guided high-power ablation pattern. High-power (45 W; temperature 43°C; saline irrigation 15 ml/min) ablation was applied until the AI target was achieved (Figures 1A,B). The VisiTag module was used during the ablation (catheter stability range of motion of 3 mm for 5 s; force ranges 10–15 g for 70% of the time; Biosense Webster). Serious procedure-related complications were defined as cardiac tamponade, thromboembolic complications, phrenic palsy, PV stenosis, or atrioesophageal fistula.

### Single-ring isolation of pulmonary veins and PLA

**Figure 2:**
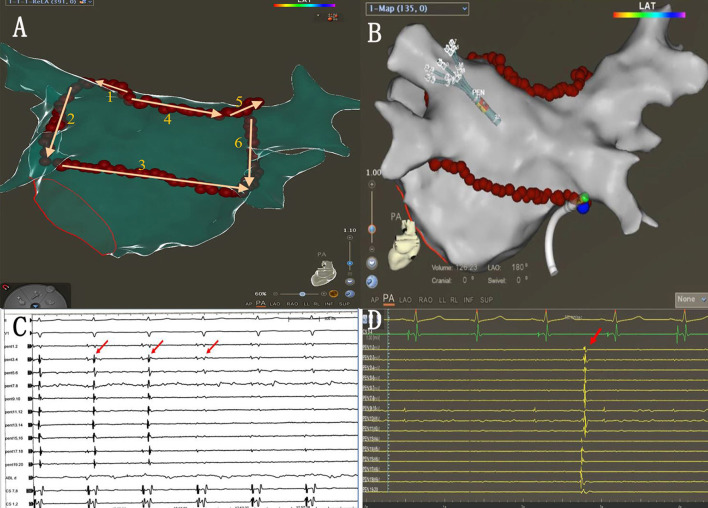
**(A)** Sequence of linear ablation. **(B)** The ablation line was completed at the bottom of the right pulmonary veins (blue dots indicate the last ablation point). **(C)** When the ablation line was completed, PVI was also completed (red arrows). **(D)** Autonomic potentials recorded with PENTARAY^®^ catheter within the ablation line.

**Figure 3:**
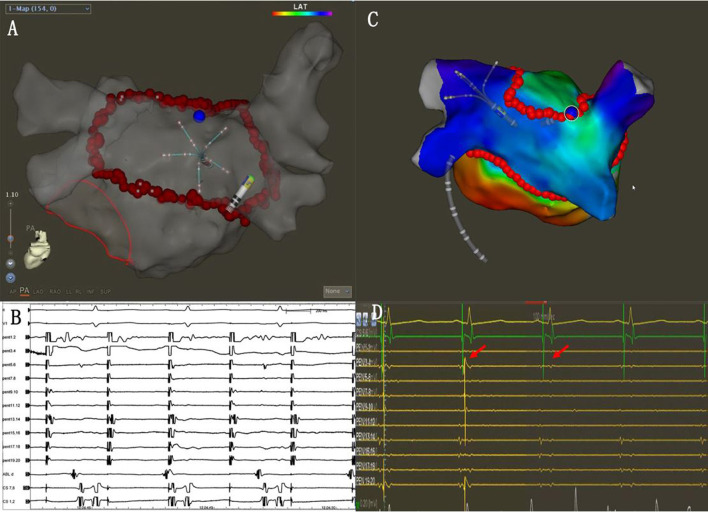
**(A)** The PENTARAY^®^ catheter was placed at the LAPW. **(B)** Efferent blocks could be recorded when pacing at the LAPW. **(C)** Activation mapping when pacing at the distal CS (CSd). The gap was located along the ablation line (blue dots). **(D)** Additional ablation was performed at the gap, and the potential was eliminated (red arrows).

The single-ring isolation line was divided into six segments as follows: the roof of the left PV, the roof line, the roof of the right PV, the anterior wall of the right PV, the ridge, and the inferior line. The ring was started from the roof of the left PV, followed by the ablation of the ridge, the inferior line, the roof line, the roof of the right PV, and the anterior wall of the right PV. The ablation line was completed at the bottom of the right PV (Figures 2A,B). The AI (ablation index) target was set to 450 for the LA roof and the posterior inferior line and 500 for the ridge and the anterior of the right PVs. The procedural endpoint of SRI was defined as follows: (1) PVs and LAPW potential disappeared or dissociated potentials as coronary sinus (CS) pacing (Figures 2C,D) and (2) pacing at the LAPW to identify the LAPW exit block (Figures 3A,B). If the PVs and PW were not isolated when the ablation line was completed, activation mapping was used to locate the gaps. Pacing at the distal CS/proximal CS (CSd/CSp) was performed to locate the gaps, and additional ablation was performed until the PVs and LAPW isolation was achieved (Figures 3C,D, Supplementary Video 1).

### SVC isolation

**Figure 4:**
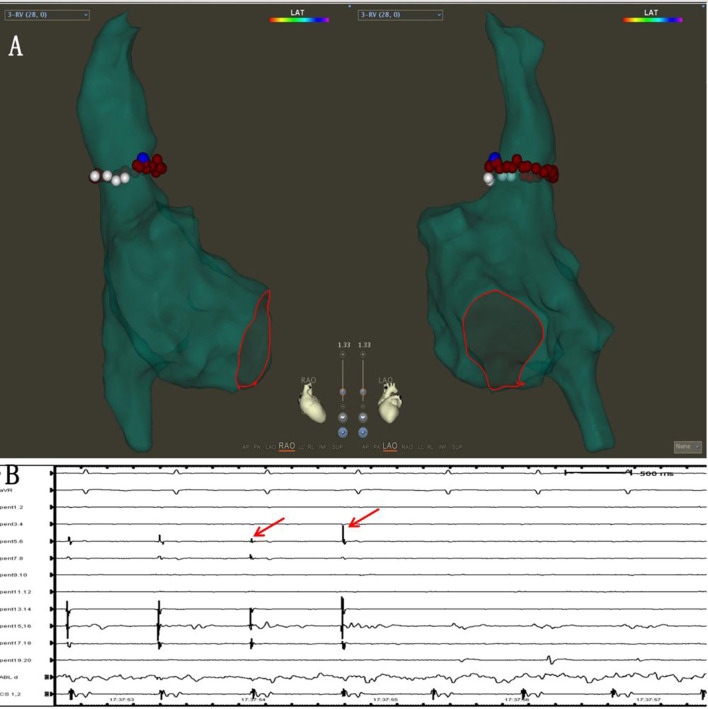
**(A)** Pacing was performed around SVC, and the phrenic nerve (PN) capture sites were marked (white dots). The marked PN capture sites (white dots) were skipped during the SVCI ablation (red dots). **(B)** The SVC potentials disappeared during the ablation (red arrows).

Before the SVCI, the PENTARAY^®^ catheter was placed at the SVC right atrium (RA) junction guided by SVC angiography. The regions of SVC were divided into four segments as follows: anterolateral, lateral, posterolateral, and septal. The order of ablation was the septal, anterolateral, and posterolateral. High-output pacing (output 10–15 V; pulse width 2–4 ms) was performed around SVC, and the phrenic nerve (PN) capture sites were marked before ablation. Automated lesion annotation was performed using the VisiTag module. Ablation power was 45 W at septal and 25 W at lateral, and the AI target was set to 400. Ablation was terminated immediately at the time of detectable reduction in diaphragmatic contraction strength. The marked PN capture sites were skipped to avoid PN injury (Figures 4A,B). The endpoint of SVCI was the elimination of SVC sleeve potentials or complete conduction block of SVC potentials to the right atrium.

### Postprocedural management and follow-up

All patients received proton pump inhibitors (PPI) (80 mg per day orally) for 4 weeks after ablation. Use of anti-arrhythmic drugs during the blanking period was allowed, but discontinuation after the blanking period was strongly recommended.

After the index ablation procedure, follow-up at 1, 3, 6, 12, and 24 months and beyond was scheduled for each patient. Briefly, a 7–14 day Holter ECG was acquired at 3, 6, 12, and 24 months after the operation. In addition to the scheduled follow-up, telephone follow-up has been carried out by dedicated follow-up commissioners, and patients were strongly recommended to visit the clinics for Holter ECG monitoring if they felt palpitation, shortness of breath, or irregularity of pulses. Arrhythmia recurrence was defined as any AF/AT episode lasting over 30 s after the 90-day blanking period.

### Statistical analysis

Continuous data were given as mean ± SD. Categorical variables were given as absolute numbers with percentages. Freedom from atrial arrhythmia recurrence during follow-up was analyzed using survival analysis, including Kaplan–Meier estimates and Cox regression. Odds ratios were presented with 95% Cl. A *P*-value ≦ 0.05 was deemed statistically significant. All statistical analyses were completed using SPSS (Version 26, IBM Corp. Armonk, NY, USA).

## Results

### Patient characteristics

From May 2019 to March 2021, a total of 60 patients were included. Baseline characteristics are summarized in Table 1. The mean age was 60 ± 9 years, and 41 (68.3%) of them were men. The mean left atrial diameter was 38.6 ± 4.9 mm. A CHA2DS2-VASc score below 3 was obtained on 35 out of 60 (58.3%) patients. All patients were treated with oral anticoagulants (NOACs), and 45 out of 60 (75%) patients were treated with antiarrhythmic drug (AAD) therapy after index ablation during the blanking period. All other patients had an intolerance or contraindication to chronic AAD therapy.

**Table 1:** Patient baseline characteristics.

**Clinical characteristics**	**Value**
Age (years)	60 ± 9
Male gender *n* (%)	41 (68.3%)
Hypertension *n* (%)	39 (65%)
Diabetes mellitus *n* (%)	13 (21.7%)
Coronary artery disease *n* (%)	14 (23.3%)
History of stroke or TIA *n* (%)	26 (43.3%)
CHA2DS2-VASc score	
0	11
1	13
2	11
3	10
≥4	15
Echocardiography parameters	
Left ventricular ejection fraction (%)	57 ± 4
Left atrial diameter (mm)	38.6 ± 4.9
Drug therapy	
Oral anticoagulant therapy	60 (100%)
AAD therapy	45 (75%)

Values are presented as mean ± SD or as *n* (%). ADD, antiarrhythmic drug; TIA, transient ischemic attack.

### Clinical outcome

The procedural data are shown in Table 2. The total procedural time was 109.9 ± 52.9 m and the fluoroscopy time was 29.6 ± 14.5 m. Acute SRI and SVCI rates were 100 and 97%, respectively.

**Table 2:** Procedural characteristics and complication.

**Clinical characteristics**	**Value**
Procedural time (min)	109.9 ± 52.9
Fluoroscopy time (min)	29.6 ± 14.5
Acute PVI success rate *n* (%)	60 (100%)
SVCI success rate *n* (%)	58 (97%)
Complication	
Diaphragmatic paralysis	0
Stroke	1
Other complication	0
Cardiac tamponade	0
Hematoma	1
Femoral pseudoaneurysm	0

SVCI, superior vena cava isolation; PVI, pulmonary veins isolation.

### Single-ring isolation

**Figure 5:**
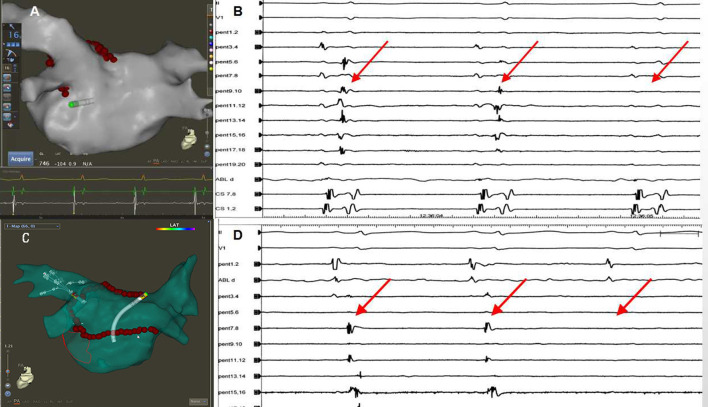
The left PV isolation was achieved with ablation line sparing. **(A)** The left PV isolation was achieved after the ridge and roof lines were completed. **(B)** The elimination of the left PV potential (red arrow) when the ridge line and the left PV roof lines were completed. **(C)** The superior and inferior left PVs were isolated when ablating at the roof near the right superior PV after the left ridge line and the inferior line were completed. **(D)** The elimination of the left PV potentials (red arrow) when ablating at the roof near the right superior PV.

In 41 out of 60 patients, one-pass SRI was achieved. In 37/41 of patients, SRI of PVs and LAPW were achieved when the ablation line was completed at the bottom of the right PV. In 4 our of 41 cases, the left PV potential was eliminated with ablation line sparing. To be specific, in 2 patients, left PVs were isolated after the ridge and roof ablation lines were completed. In 2 (5%) patients, left PVs were isolated when ablating at the right posterior-superior right PVs, after the left ridge line, the roof line, and the inferior line were completed (Figure 5).

In the remaining 19 out of 60 (31.7%) patients, additional ablation was necessary to achieve PVI and PWI. Gaps were defined as the earliest activation points on the ablation line or within the circle when pacing was at CS. The most common gaps were located on the roof line near the right superior pulmonary vein (RSPV), accounting for 10 (16.7%) gaps. At the ridge of the left PVs, two gaps (3.3%) were located, and at the middle of the inferior line, one gap (1.7%) was located. In 6 (10%) patients, the gaps were within the circle, over 10 mm away from the ablation lines, suggesting the conduction of epicardial insertions.

Superior vena cava isolation

Isolation of SVC was attempted in all patients during the index ablation. In all patients, the PNs were captured, and the capture points were marked. In 58 (96.7%) patients, the SVCI was achieved. A total of 10 SVCIs were completed at the septal wall, 12 at the anterolateral and septal walls, 14 at the posterolateral and septal walls, and 22 at the anterolateral and septal walls and the posterolateral wall. The SVCI was not completed in 2 cases due to concerns about the phrenic nerve (PN) injury. After isolation of SVC, pacing at the SVC markers was repeated to check whether PN was injured. After SVCI, one patient had decreased diaphragmatic contraction, which was restored 15 min later.

### Complications

During the follow-up period, one patient had an ischemic stroke but recovered fully after medical treatment. Two patients had minor groin hematomas. There were no deaths or other severe complications like cardiac tamponade, PV/SVC stenosis, the sinoatrial node, phrenic nerve injury, major bleeding, PV stenosis, and atrioesophageal fistula.

### Follow-up

**Figure 6:**
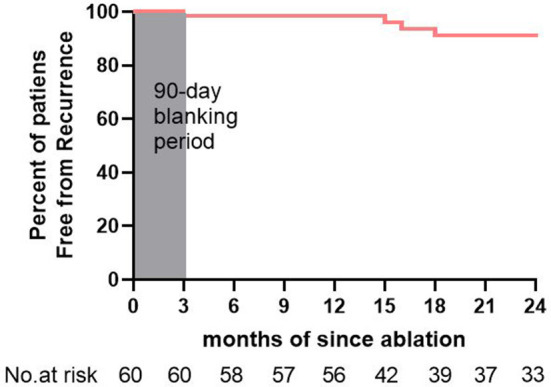
Survival curve after the single catheter ablation procedure.

The average follow-up period was 20 ± 7 months. There were no deaths or any stroke/TIA events. Notably, 45 (75%) patients were taking AAD, and all patients were taking NOACs. After their *de novo* ablation, 55 (91.7%) patients remained free from >30 s AF/AT (Figure 6). Transient AF or AT episodes lasting less than 30 s were noted in 3 (5%) patients. Recurrences were recorded in 5 (8%) patients, of which 4 had paroxysmal AF and 1 had organized atrial tachycardia. Among the 5 patients, 2 underwent redo ablation for paroxysmal AF episodes. In the redo ablation, the conduction of both the PVs and the LAPW had been recovered. In one patient, a gap was located at the ridge of the left PVs. In the other patient, the gap was located at the bottom of the right inferior PV. SVC potential had not recovered in these 2 patients. In addition, the 2 patients were continuously followed after their redo ablations, and no recurrence was recorded. Altogether, 57 (95%) patients remained free of AF/AT episodes lasting over 30 s after multiple ablations.

## Discussion

### Main findings

This is the first attempt to an investigation of the ablation strategy using SRI combined with SVCI for PAF. SRI plus SVCI could be completed safely. It shows potential for improving clinical outcomes in contrast to previous studies (14, 15). SRI is particularly suitable for patients with the common ostium of the inferior pulmonary veins.

### Single-ring isolation of pulmonary veins

Some previous studies reported the ablation technique of SRI for paroxysmal and persistent AF (6, 16) and that SRI could improve the success rate (7, 17). However, it also had the following disadvantages: the single-ring ablation line was relatively long and it was difficult to isolate the PVs at the initial ablation attempt. In this study, we used AI-guided high-power ablation to ensure durable lesion formation, considering the VISITAG SURPOINT was a lesion-quality marker that improved the outcomes of AF ablation (18, 19). At the same time, high-power ablation has also been proven to be effective in PVI ablation (20). In our study, one-pass single-ring PVI could be completed in most of the patients without additional ablation (41/60, 68%). We postulated that high power could be effective in achieving the transmural tissue injury and that AI-guided ablation could monitor the stability of the catheters, both of which could improve the success rate of SRI.

We would have expected that the four pulmonary veins would be isolated simultaneously after the ablation line was completed. However, in four cases, before the ablation line was completed, the left pulmonary vein potential disappeared. The left pulmonary vein may have a less fibrous connection with the posterior wall of the left atrium as well as the right PV.

For the patients whose PVs and LAPW potential were not eliminated after the ablation lines were completed, activation mapping under CS pacing was effective in locating the gaps. In our study, 19 out of 60 patients needed additional ablation to complete the electrical isolation. Gaps at the roof line close to the right superior PV were located in 10 out of 19 cases. The relatively thicker atrial muscle and more atrial fibers crossing this region may have contributed to this result (21, 22).

**Figure 7:**
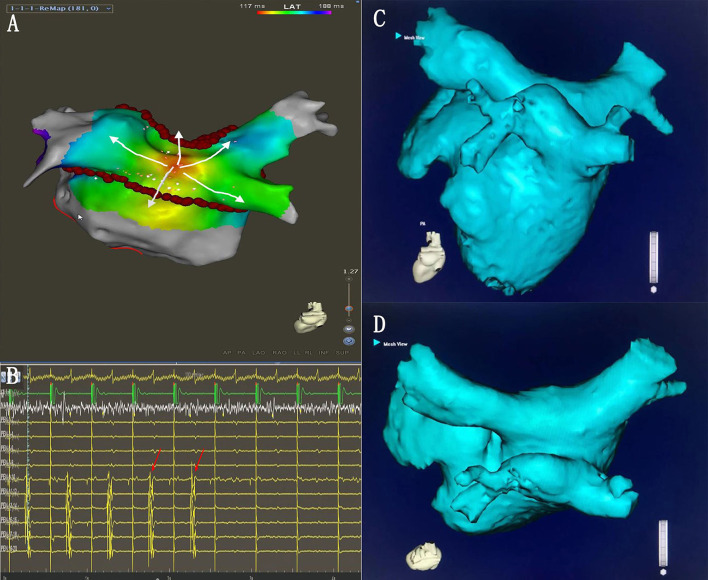
**(A)** Activation mapping under the CS pacing. The earliest activation site was located on the LAPW (white arrows). **(B)** During the additional ablation at the LAPW earliest activation point, the potential disappeared. **(C,D)** The common ostium of inferior pulmonary veins of the anatomical models.

On the LAPW wall, 6 gaps were located far from the ablation lines (Figures 7A,B, Supplementary Video 1). Recent research showed that the septopulmonary bundle (SPB) mediates epicardial conduction across the roof line to the LAPW (23–25). When the PVs and LAPW potential were not isolated, epicardial connections on the LAPW should also be taken into account.

### The common ostium of inferior pulmonary veins (PVs)

The common ostium of the inferior pulmonary veins (COIPV) is an uncommon anatomical variation of the PVs (Figures 7C,D), as reported in previous studies (26, 27). It is rare but challenging for PVI ablation (28, 29). Yu et al. (28) used the “tri-circle” strategy to finish the circumferential PV isolation, and Li et al. (30) used the second-generation cryoballoon at the ostium of the left and right inferior pulmonary veins. The above methods inevitably increase the ablation dosing of LAPW. SRPVI could isolate the PVs with unusual PV anatomical variation (common left PV ostium, common right PV ostium, COIPV, and supernumerary PVs), without increasing the ablation dosing on the LAPW.

### Electrical isolation of the SVC

Previous studies showed that additional SVCI could improve the success rate of PAF (10, 12, 31). However, SVC ablation might be related to complications such as PN injury and sinus node injury (32, 33). To prevent the occurrence of PN injury, the electrical isolation of the SVC can be achieved by segmental ablation, and PN pacing was required during ablation or before ablation. It was very important to precisely locate the junction according to SVC angiography as well as to maximize the marked diaphragmatic capturing sites with high output pacing at the lateral segment of SVC. Although we safely accomplished most of the SVCI, there was still one patient who had a transient decrease in diaphragmatic contraction after ablation. Ablation near the site of diaphragmatic stimulation requires great care, although we observed reduced power consumption in this area.

## Conclusion

Ablation strategy (SRI combined with SVCI) appears to be an effective and safe strategy for paroxysmal atrial fibrillation. It is a promising ablation strategy for patients with the common ostium of inferior PVs.

### Limitations

This was an uncontrolled retrospective single-center study with a small sample size, and prospective randomized controlled studies and future studies with larger samples are needed to confirm its validity.

## Data availability statement

The raw data supporting the conclusions of this article will be made available by the authors, without undue reservation.

## Ethics statement

The studies involving human participants were reviewed and approved by the Ethical Committee of Wuhan Asian Heart Hospital (2022-B038). The patients/participants provided their written informed consent to participate in this study.

## Author contributions

XL and YC: data collation, statistical analysis, and paper writing. JZ, GC, CD, and CT: research guidance and paper revision. All authors contributed to the article and approved the submitted version.
